# Load-distributing band improves ventilation and hemodynamics during resuscitation in a porcine model of prolonged cardiac arrest

**DOI:** 10.1186/1757-7241-20-59

**Published:** 2012-09-01

**Authors:** Shuo Wang, Jun-Yuan Wu, Chun-Sheng Li

**Affiliations:** 1 Department of Emergency Medicine, Beijing Chaoyang Hospital, Affiliated to Capital Medical University, Chaoyang District, Beijing, 100020, China

**Keywords:** Cardiopulmonary resuscitation, Rescue breathing, Load-distributing band, Coronary perfusion pressure, Dead space

## Abstract

**Background:**

The use of mechanical cardiopulmonary resuscitation (CPR) has great potential for the clinical setting. The purpose of present study is to compare the hemodynamics and ventilation during and after the load-distributing band CPR, versus the manual CPR in a porcine model of prolonged cardiac arrest, and to investigate the influence of rescue breathing in different CPR protocols.

**Methods:**

Sixty-four male pigs (n = 16/group), weighing 30 ± 2 kg, were induced ventricular fibrillation and randomized into four resuscitation groups: continuous load-distributing band CPR without rescue ventilation (C-CPR), load-distributing band 30:2 CPR (A-CPR), load-distributing band CPR with continuous rescue breathing (10/min) (V-CPR) or manual 30:2 CPR (M-CPR). Respiratory variables and hemodynamics were recorded continuously; blood gas was analyzed.

**Results:**

Tidal volume produced by compressions in the A-, C- and V-CPR groups were significantly higher compared with the M-CPR group (all p < 0.05). Coronary perfusion pressure of the V-CPR group was significantly lower than the C-CPR group (p < 0.01), but higher than the M-CPR group. The increasing of lung dead space after restoration of spontaneous circulation was significantly greater in the M-CPR group compared with the A-, C- and V-CPR groups (p < 0.01). Blood pH gradually decreased and was lower in the M-CPR group than that in the A-, C- and V-CPR groups (p < 0.01). PaO_2_ of the A-, C- and V-CPR groups were significantly higher and PaCO_2_ were significantly lower compared with the M-CPR (both p < 0.05). Cerebral performance categories were better in the A-, C- and V-CPR groups compared with the M-CPR group (p < 0.0001).

**Conclusions:**

The load-distributing band CPR significantly improved respiratory parameters during resuscitation by augmenting passive ventilation, and significantly improved coronary perfusion pressure. The volume of ventilation produced by the load-distributing band CPR was adequate to maintain sufficient gas exchange independent of rescue breathing.

## Introduction

Restoration of spontaneous circulation (ROSC) is the basic aim of cardiopulmonary resuscitation (CPR). Many studies have discussed the most appropriate CPR protocols to provide the greatest ROSC [1-3]. The 2010 AHA guidelines emphasized the alternation of rescuers in order to avoid fatigue and maintain effective CPR. Some technological equipment, such as the Q-CPR developed by Philips Medical Systems, has been used in the clinical setting in order to maintain the quality of CPR. A recent study reported the Q-CPR to be of great benefit in providing efficient CPR [2]. Moreover, a portable battery-driven electromechanical device (AutoPulse™, Zoll Medical Corporation, Chelmsford, MA) has been developed that is capable of compressing the anterior chest via a load-distributing band (LDB). This system offers a substitute in providing sustainable and proficient CPR while avoiding rescuer’s fatigue. Studies investigating this device have reported that it could provide superior hemodynamics, cerebral and myocardial blood flow when compared to pneumatic or manual chest compression in a porcine model and human investigations [4-7]. Otherwise, negative result of LDB CPR was also arisen from the Circulation Improving Resuscitation Care (CIRC) trial [8].

Former studies have confirmed that at the time of compressions, recoil of the thoracic cage could generate a little ventilation [9-11]. This ‘passive ventilation’ may improve gas exchange across the lung during resuscitation and be good to hemodynamics. This might make the rescue breathing less important and support the strategy of continuous compressions without rescue breathing. However few studies have yet focused on whether CPR produced by a mechanical device can improve passive ventilation. The present prospective randomized animal study sought to compare the hemodynamics and ventilation during and after the LDB CPR, versus the manual CPR, to investigate the influence of rescue breathing in the different CPR protocols.

## Materials and methods

### Animal preparation

This study was conducted with the approval of the Capital Medical University Institutional Animal Care Committee.

Sixty-four male domestic pigs (10–12 weeks, 30 ± 2 kg) were used in the current study. Animals were fasted overnight and had free access to water. After pretreatment with intramuscular administration of midazolam 0.2 mg/kg, anaesthesia was induced by ear vein injection of propofol (1.0 mg/kg) and animals were maintained in a surgical plane of anaesthesia with continuous intravenous infusion of pentobarbital (8 mg/kg per hour). All animals were intubated with a cuffed 6.5 mm endotracheal tube, and ventilated using a volume-controlled ventilator (PB-7200, Nellcor Puritan Bennett Inc., Boulder, CO) with an initial tidal volume (TV) of 8 ml/kg and a respiratory frequency of 15/min with room air. End-tidal PCO_2_ (EtPCO_2_) was measured by an in-line infrared capnograph (CO_2_SMOplus monitor, Respironics Inc., Murrysville, PA). Respiratory frequency was adjusted to maintain EtPCO_2_ between 4.7 and 5.3 kPa before inducing cardiac arrest. Room temperature was maintained at 26°C. A Swan-Ganz catheter (7F, Edwards Life Sciences, Irvine, CA) was flow-directed into the pulmonary artery from the right femoral vein for measurement of right atrial pressure (RAP) and cardiac output (CO). An angiographic catheter was inserted from the femoral artery into the aortic arch for reference blood samples and measuring aortic pressure (AOP). The electrocardiogram (ECG) and all hemodynamic parameters were visualized using a HP monitor (M1165, Hewlett-Packard, Palo Alto, CA). An electrode catheter was inserted into the left external jugular vein to induce ventricular fibrillation by a programmed electrical stimulation instrument (GY-600A, Huanan Instrument Company, Kaifeng, Henan, China).

### AutoPulse™ device

The AutoPulse™ system is a portable chest compression device constructed around a backboard, which uses a motor to retract a LDB automatically. When the band is enclosed around the chest and the device started, the band automatically tightens to fit the diameter of thoracic cage and the motor tightens and loosens the band periodically. The AutoPulse™ is programmed to provide a constant 50 mm reduction in the anterior-posterior dimension of the individual patient’s chest. The compression rate is maintained at 80 ± 5/min. The period’s proportion of compression/unloading is 1/1. The mode can be selected to produce continuous compressions or 30:2 CPR. In the 30:2 CPR mode, compressions are halted for 3 s every 30 compressions to allow two rescue ventilations to be given to the patient.

The AutoPulse™ device used in the present study comprised a LDB that was scaled down from the original device designed for human use. This porcine-sized LDB allowed an analogous proportion of the porcine thorax to be compressed by the band.

### Quality control of CPR

The quality of manual chest compressions was determined by a HeartStart MRx Monitor/Defibrillator with Q-CPR technology (Philips Medical Systems, Best, Holland). Objective measurements were determined using a compression sensor, which measured the acceleration of the chest during chest compressions. An algorithm in the monitor converted the acceleration to compression depth. A characteristic waveform was shown on the screen to indicate the change in diameter of the thoracic cage and the compression rate. Two horizontal lines were drawn on the top of the wave indicating the target zone to aid the rescuer achieving the good compression depth of 50 ± 1 mm in accordance with the AHA guidelines.

### Experimental protocol

Forty-five minutes after the completion of all surgery, baseline values were obtained. VF was induced by programmed electrical stimulations using mode S1S2 (300/200 ms), eliciting 40v in 8:1 proportion and −10 ms step length [12]. When ventricular fibrillation was confirmed by ECG with the presence of profound hypotension, ventilation was stopped and the ventilator was disconnected from the endotracheal tube. After 4 min of untreated ventricular fibrillation, animals were randomized to receive either continuous LDB CPR without rescue breathing (C-CPR), LDB 30:2 CPR (A-CPR), LDB CPR with continuous rescue breathing (10/min) (V-CPR) or manual 30:2 CPR (M-CPR) (n = 16 piglets for each group). Compressions were performed 100 times per minute in the M-CPR groups. The rescue breathing conducted on the A-CPR and M-CPR groups were performed twice at the 3 s pause between two 30-compression cycles using a bag respirator with 300 ml of room air. In the V-CPR group, rescue breathing was performed 10/min continuously with the bag respirator of 300 ml room air. In the M-CPR group, compressions were performed by two experienced researchers, who alternated every 2 min. The quality of M-CPR, compression rates and defibrillation shocks were performed by a HeartStart MRx Monitor/Defibrillator, which was open to the resuscitators for optimizing the M-CPR. After 12 min of chest compressions, defibrillation shocks were applied at 150 J with Smart Biphasic wave for the first attempt. If the first defibrillation was unsuccessful, epinephrine (0.02 mg/kg) was administered intravenously, followed by 2 min of CPR. After each 2 min of CPR, a 10 s pause was interjected to prepare for the next defibrillation attempt, and the rhythm was analysed. ROSC was defined as more than 10 consecutive minutes of maintaining systolic blood pressure above 50 mmHg. Animals with no ROSC after 4 attempts of defibrillation were pronounced dead. After successful resuscitation, animals were continuously ventilated and underwent 6 h of intensive care where Ringer's solution (20 ml/kg/h) was administered. With the exception of one jugular vein sheath that was used for fluid administration, all other vascular sheaths as well as the endotracheal tube were removed. Animals were allowed to recover from anaesthesia, and placed in an observation cages for monitoring over the ensuing 24 h. Water was given during the observation period. Neurological outcomes of animals were blinded evaluated according to the porcine cerebral performance categories (CPCs) at 24 h after ROSC as previously described [13]. Pathological examination was performed on all animals to evaluate the possibility of rib or sternal fracture as well as gross injury of the lungs or liver.

### Measurements

RAP, AOP and ECG were continuously measured and recorded throughout the resuscitation period. Coronary perfusion pressure (CPP) was defined as the difference between the diastolic aortic pressure (AOD) and the diastolic right atrial pressure (RAD).

Arterial blood samples for blood gas analyses (GEM Premier 3000, Instrumentation Laboratory, Lexington, MA) were drawn at baseline, 4, 8, and 12 min of CPR and 1 h after ROSC. Saline (4°C) was injected into the right atrium at baseline and 1 h after ROSC through the Swan-Ganz catheter to determine CO by the transpulmonary thermodilution method as previously described [14]. Continuous real-time respiratory variables (gas flow and volume, airway pressure and EtPCO_2_) were measured using a calibrated CO_2_SMOplus monitor. Data were downloaded via a RS232 connection to a portable computer. Dead space (VD) was determined using the following alveolar gas equation with arterial blood gas measurements at baseline and after 1 h of ROSC.

(1) $$VD=TV\times \frac{PaCO_{2}-EtPCO_{2}}{PaCO_{2}}$$

VD was subtracted from TV to calculate the alveolar minute volume (MValv).

### Statistical analysis

Data were analysed by Statistical Package for the Social Sciences (SPSS) 17.0 statistics software (SPSS Inc., Chicago, IL) and reported as the mean ± SD. Discrete variables, including the number of ROSC and survival, were compared with a Fisher’s exact test. Because of the correlations among different time points of continuous variables, including hemodynamics, blood gases and respiratory parameters, the differences among groups were detected by multivariate ANOVA, in which all variables were considered in a multivariate model and tested significance. If significant differences were found, post hoc with least-significant difference test were performed to detect specific differences between each group. The p < 0.05 was regarded as being statistically significant.

## Results

### Outcomes

15/16 (93.8%) piglets of the C-CPR and A-CPR group and 13/16 (81.3%) of the V-CPR group had ROSC, but only 11/16 (68.8%) of the M-CPR group had ROSC. No significant difference of ROSC was found between each two groups. The 4 h survival rates were also found to be higher in the C-, A- and V-CPR group compared to the M-CPR group, while it was not differ in the C-, A- and V-CPR groups. CPC was significantly higher in the M-CPR group compared to the three LDB CPR groups (both p < 0.0001). No significant difference in CPC was found among the A-, C- and V-CPR group (Table 1). No rib fracture was identified in animals received LDB CPR; on the contrary, 8/16 (50.0%) piglets in the M-CPR group were found to have rib fractures and 3/16 (18.8%) of the M-CPR group exhibited lung injury.

**Table 1:** Outcomes following CPR

**Outcomes**	**A-CPR (n = 16)**	**C-CPR (n = 16)**	**V-CPR (n = 16)**	**M-CPR (n = 16)**
ROSC	15	15	13	11
Shocks before ROSC	1.80 ± 0.86	1.93 ± 0.96	1.92 ± 0.74	2.45 ± 1.37
Duration of CPR before ROSC (s)	823 ± 109	839 ± 121	836 ± 108	871 ± 136
4h survival	15	15	13	10
24h survival	14	14	13	10
CPC at 24h	1.62 ± 0.77 ^a^	1.50 ± 0.65 ^a^	1.53 ± 0.77 ^a^	3.82 ± 0.87

CPR: cardiopulmonary resuscitation; A-CPR: load-distributing band 30:2 CPR; C-CPR: continuous load-distributing band CPR without rescue ventilation; V-CPR: continuous load-distributing band CPR with continuous rescue ventilation; M-CPR: manual 30:2 CPR; ROSC: restoration of spontaneous circulation; CPC: cerebral performance categories.

a. p < 0.0001, vs. M-CPR group.

### Respiratory parameters

**Figure 1:**
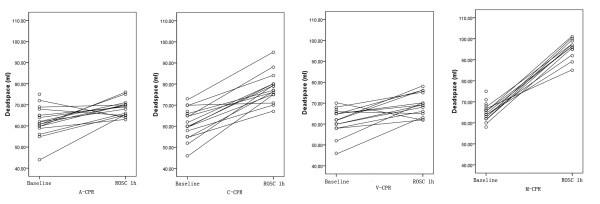
**Increase in dead space after 1 h of restore of spontaneous circulation.** Dead space increased significantly within each treatment group, most dramatically in the M-CPR group. The increasing of dead space in each group was found significantly differ from each other (all p < 0.01). CPR: cardiopulmonary resuscitation; A-CPR: load-distributing band 30:2 CPR; C-CPR: continuous load-distributing band CPR without rescue breathing; V-CPR: continuous load-distributing band CPR with continuous rescue breathing; M-CPR: manual 30:2 CPR; ROSC: restoration of spontaneous circulation.

The mean value of VD in A-, C-, V- and M-CPR groups at baseline were 62.3 ± 7.3 ml, 61.5 ± 7.2 ml, 61.5 ± 6.1 ml and 65.4 ± 4.2 ml respectively; no significant difference was found between each two groups. The VD of each group significantly increased to 68.5 ± 3.9 ml, 78.2 ± 7.0 ml, 69.2 ± 5.4 ml and 95.0 ± 4.8 ml respectively after 1 h of ROSC (all p < 0.05). The increasing of VD was 7.8 ± 6.0 ml, 17.1 ± 7.1 ml, 8.6 ± 6.0 ml and 30.7 ± 6.0 ml respectively. Notably, the M-CPR group displayed the greatest increase in VD above the other groups and C-CPR group displayed the greatest increasing among the three LDB CPR groups (Figure 1).

**Figure 2:**
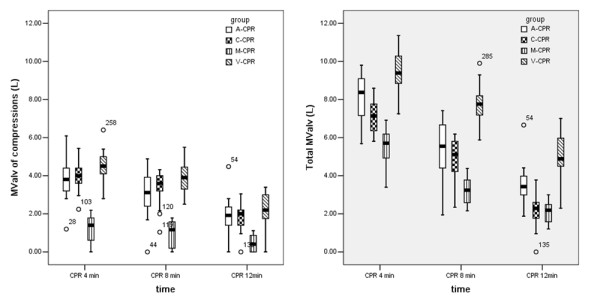
**Total alveolar minute volume (MValv) and MValv of compressions during cardiopulmonary resuscitation in each group.** Load-distributing band compressions produced significantly greater ventilation compared to manual compressions at each time point. The MValv of compressions decreased progressively in all groups, most notably in the M-CPR group where reductions in MValv was significantly greater than A-, C- and V-CPR groups (both p < 0.0001). Total MValv was also reduced because of the reduction in MValv of compressions and gasps. CPR: cardiopulmonary resuscitation; A-CPR: load-distributing band 30:2 CPR; C-CPR: continuous load-distributing band CPR without rescue breathing; V-CPR: continuous load-distributing band CPR with continuous rescue breathing; M-CPR: manual 30:2 CPR; ROSC: restoration of spontaneous circulation. Total MValv = MValv of compression + MValv of gasps + MValv of manual ventilation.

All CPR protocols used in the current study could produce ventilation by compression; the generated volume was higher than the VD in the majority of animals. The ventilation volume produced by LDB compressions was significantly greater than manual compressions at each time point (p < 0.05). In 2/16 (12.5%) piglets of the M-CPR group, compressions could not produce a ventilation volume greater than the VD at CPR 4 min. This phenomenon was not observed in the LDB CPR groups. In all groups, the volume produced by compressions, namely MValv of compressions, decreased progressively, especially in the M-CPR group (Figure 2). So the piglets number of not producing a ventilation volume greater than the VD increased to 1/16 (6.3%), 2/16 (12.5%), 2/16 (12.5%) and 4/16 (25.0%) in the A-, C-, V- and M-CPR group respectively at CPR 12 min.

Gasps were observed in all animals of all groups and decreased with the duration of the compressions. Total MValv was calculated as the sum of the MValv of compressions, gasps and rescue breathing. Because of the reduction in MValv of compressions and gasps, the total MValv was also reduced (Figure 2).

### Hemodynamics

**Figure 3:**
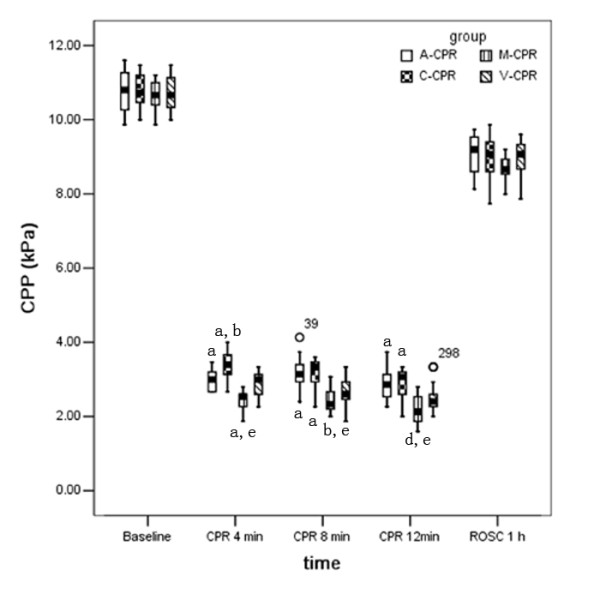
**CPP decreased progressively during cardiopulmonary resuscitation across all groups.** It was significantly better following LDB CPR compared with manual’s. The V-CPR group displayed lower CPP than the C-CPR group, which demonstrated that rescuing breathing simultaneous with compressions might adverse to hemodynamics. a. p < 0.01, vs. M-CPR group; b. p < 0.01, vs. A-CPR group; c. p < 0.05, vs. M-CPR; d. p < 0.05, vs. A-CPR group; e. p < 0.01, vs. C-CPR group. CPR: cardiopulmonary resuscitation; A-CPR: load-distributing band 30:2 CPR; C-CPR: continuous load-distributing band CPR without rescue ventilation; V-CPR: continuous load-distributing band CPR with continuous rescue ventilation; M-CPR: manual 30:2 CPR; ROSC: restoration of spontaneous circulation; CPP: coronary perfusion pressure.

AOD and CPP decreased progressively during CPR; however, they were maintained at significantly elevated levels following A- and C-CPR when compared with M-CPR (p < 0.05). In LDB CPR groups, V-CPR produced the lowest AOD and CPP; significant differences were found between V-CPR and other two LDB CPR groups. No significant differences of AOD, CPP or CO after 1 h of ROSC were found among the four groups (Table 2, Figure 3).

**Table 2:** Hemodynamics and blood gas analyses

	**Baseline**	**CPR 4 min**	**CPR 8 min**	**CPR 12 min**	**ROSC 1 h**
AOD (kPa)					
A-CPR	11.2 ± 0.5	3.8 ± 0.3 ^a^	4.1 ± 0.4 ^a^	3.9 ± 0.4 ^a^	9.9 ± 0.6
C-CPR	11.2 ± 0.5	4.2 ± 0.4 ^a, b^	4.0 ± 0.3 ^a^	3.9 ± 0.4 ^a^	9.7 ± 0.6
V-CPR	11.1 ± 0.5	3.7 ± 0.3 ^a, e^	3.7 ± 0.3 ^b, c, e^	3.2 ± 0.4 ^b, e^	9.8 ± 0.6
M-CPR	11.1 ± 0.5	3.2 ± 0.3	3.2 ± 0.3	3.5 ± 0.4	9.5 ± 0.4
RAD (kPa)					
A-CPR	0.41 ± 0.13	0.80 ± 0.10	0.89 ± 0.10	1.00 ± 0.13	0.86 ± 0.11
C-CPR	0.37 ± 0.14	0.80 ± 0.10	0.84 ± 0.11	0.96 ± 0.07	0.84 ± 0.11
V-CPR	0.37 ± 0.12	0.80 ± 0.10	0.85 ± 0.11	0.95 ± 0.10	0.85 ± 0.11
M-CPR	0.39 ± 0.13	0.74 ± 0.12	0.85 ± 0.07	0.95 ± 0.11	0.80 ± 0.08
CPP (kPa)					
A-CPR	10.8 ± 0.5	3.0 ± 0.3 ^a^	3.2 ± 0.4 ^a^	2.9 ± 0.4 ^a^	9.0 ± 0.6
C-CPR	10.8 ± 0.5	3.4 ± 0.4 ^a, b^	3.2 ± 0.4 ^a^	2.9 ± 0.4 ^a^	8.9 ± 0.7
V-CPR	10.7 ± 0.5	2.9 ± 0.3 ^a, e^	2.7 ± 0.4 ^b, e^	2.5 ± 0.4 ^d, e^	8.9 ± 0.6
M-CPR	10.7 ± 0.4	2.5 ± 0.3	2.4 ± 0.3	2.2 ± 0.4	8.7 ± 0.3
CO (L/min)					
A-CPR	3.74 ± 0.17	-	-	-	2.77 ± 0.31
C-CPR	3.80 ± 0.21	-	-	-	2.88 ± 0.14
V-CPR	3.80 ± 0.13	-	-	-	2.88 ± 0.12
M-CPR	3.66 ± 0.20	-	-	-	2.77 ± 0.20
pH					
A-CPR	7.43 ± 0.04	7.46 ± 0.02 ^a^	7.44 ± 0.03 ^a^	7.30 ± 0.03 ^a^	7.37 ± 0.03
C-CPR	7.43 ± 0.04	7.44 ± 0.02 ^c^	7.41 ± 0.03 ^a^	7.29 ± 0.03 ^a^	7.36 ± 0.04
V-CPR	7.42 ± 0.03	7.44 ± 0.03 ^c^	7.41 ± 0.03 ^a^	7.28 ± 0.03 ^a^	7.36 ± 0.04
M-CPR	7.43 ± 0.04	7.42 ± 0.04	7.36 ± 0.06	7.22 ± 0.07	7.36 ± 0.02
PaO_2_ (kPa)					
A-CPR	11.6 ± 0.6	9.8 ± 0.5 ^a^	9.2 ± 0.5 ^a^	7.1 ± 0.4 ^a^	11.0 ± 0.7 ^a^
C-CPR	11.8 ± 0.7	9.8 ± 0.4 ^a^	8.9 ± 0.4 ^a^	6.9 ± 0.5 ^a^	10.5 ± 0.7 ^d^
V-CPR	11.9 ± 0.7	9.7 ± 0.4 ^a^	8.9 ± 0.4 ^a^	6.9 ± 0.5 ^a^	10.5 ± 0.8
M-CPR	11.4 ± 0.6	9.1 ± 0.9	7.7 ± 1.0	6.0 ± 0.6	10.3 ± 0.4
PaCO_2_ (kPa)					
A-CPR	5.1 ± 0.4	4.1 ± 0.6 ^a^	5.3 ± 0.6 ^a^	6.7 ± 0.4 ^a^	4.9 ± 0.2
C-CPR	5.1 ± 0.4	4.8 ± 0.4 ^b^	6.0 ± 0.4 ^b^	7.4 ± 0.6 ^b, c^	4.9 ± 0.2
V-CPR	5.3 ± 0.3	4.0 ± 0.2 ^a, e^	5.3 ± 0.4 ^a, e^	6.8 ± 0.5 ^a, e^	5.0 ± 0.3
M-CPR	4.9 ± 0.3	4.8 ± 0.5	6.4 ± 0.3	8.0 ± 0.8	4.8 ± 0.1

A multivariate analysis of variance revealed significant differences in AOD, CPP, PaO_2_ and PaCO_2_ during CPR at each time point between groups having undergone load-distributing band CPR and manual CPR. Changes over time were determined by ANOVA with repeated measures. Significant differences in parameters between groups were determined using a multivariate ANOVA.

a. p < 0.01, vs. M-CPR group; b. p < 0.01, vs. A-CPR group; c. p < 0.05, vs. M-CPR; d. p < 0.05, vs. A-CPR group; e. p < 0.01, vs. C-CPR group.

CPR: cardiopulmonary resuscitation; A-CPR: load-distributing band 30:2 CPR; C-CPR: continuous load-distributing band CPR without rescue ventilation; V-CPR: continuous load-distributing band CPR with continuous rescue ventilation; M-CPR: manual 30:2 CPR; AOD: diastolic aortic pressure; RAD: diastolic right atrial pressure; CPP: coronary perfusion pressure.

### Blood gas analysis

pH and PaO_2_ gradually decreased, and PaCO_2_ increased over the course of CPR, which demonstrated a slow deterioration of metabolism. Comparing LDB CPR groups to M-CPR, pH did not significantly differ; however, PaO_2_ was significantly higher and PaCO_2_ was significantly lower in animals underwent LDB CPR. At 12 min of CPR, acidosis was observed in all groups (Table 2).

## Discussion

The most imperative finding of current study was that LDB CPR could generate significantly greater ventilation than M-CPR. Increased ventilation volume should enhance gas exchange during CPR, which was confirmed in observing better changes in PaO_2_ and PaCO_2_ of LDB CPR groups. Even the porcine and human hemoglobin have different chemical structures and properties, the relations between PaO_2_ and oxygen carrying properties are not necessarily the same as human being’s, but this did not influence our founding of comparing the LDB and M-CPR in a same model. The more ventilation could prevent alveoli collapse, and then improve lung compliance and perfusion to decrease VD. The relative less VD after CPR in the A- and V-CPR group might be this reason. On the other hand, the less VD in the LDB CPR groups might due to the bigger contact area of LDB, which made the force of compressions to thoracic cage more uniform, then lightened the lung injury induced by compressions. Both of these two reasons might be good to decrease the VD after CPR.

There has been a long-standing debate whether compressions could produce adequate passive ventilation. One animal study indicated that manual compressions could provide adequate passive ventilation [10], while a human clinical study using a mechanical chest compression device (LUCAS) found that passive ventilation was limited and the gas exchange was reduced in compression-only phase [11]. The conflicting findings of these studies seemed like due to the labile compliance of each animal’s thoracic cage and the different resuscitation methods used.

Many former studies discussed the necessity of ventilation during CPR, but the outcomes were inconsistent. Ewy GA et al. demonstrated that neurological outcome was improved by continuous compressions [15], but Yannopoulos and colleagues demonstrated that when there was no assisted ventilation during CPR, 24 h neurological outcome was significantly worse compared to those with positive pressure ventilation simultaneous with CPR in pigs [16]. The main concern of this problem is whether abolishing ventilation could still maintain sufficient gas exchange during CPR. Our former study addressed this query and found that animals displayed relatively normal global V/Q values during continuous compression without ventilation, but exhibited reduced gas exchange compared to CPR with rescue breathing [17]. The founding of the current study suggest that the LDB could significantly increase ventilation and augment resuscitation via larger and sustained compressions, maintaining elevated gas exchange, without the need for rescue breathing. Together with the three groups receiving LDB CPR exhibited the same outcomes; both indicated that rescue breathing was not required.

The compliance of M-CPR was high related with the ROSC of cardiac arrest, even Q-CPR technology has been used to optimize the quality [2]. In addition, the fatigue of rescuers inevitably influenced the quality of CPR. Mechanical device could perform standard CPR without fatigue, meanwhile hemodynamics during CPR were also improved [4,5], which has been underlined by European Resuscitation Council guidelines [18].

The outcome of the present study has shown that the LDB CPR can overcome many deleterious complications associated with M-CPR. The LDB without Q-CPR was found to produce better compressions than manual with Q-CPR. LDB CPR resulted in a higher rate of ROSC, greater 24 h survival, especially significant better CPC independent of rescue breathing. Even previous study has demonstrated the neuroprotective properties of pentobarbital [19], which could keep the relative good neurological outcome when PaO_2_ around 7.0 kPa after 12 minutes of CPR in the LDB CPR group. Whereas all of our study groups used the same dose of aesthetic, the better neurological prognosis of LDB CPR could not contribute to the pentobarbital, but the different CPR protocols. Furthermore, no trauma, including rib fractures, was induced by LDB CPR, which was almost inevitable during manual CPR. Analysis of hemodynamic parameters including AOD and CPP were also significantly better following LDB CPR compared with the manual’s. In clinic, if the using of Q-CPR is impossible, similar hemodynamic and ventilatory results or clinical outcomes might not be found, whereas because of the good effect of the Q-CPR technology to M-CPR, the difference between the LDB and M-CPR might be more significant.

Both LDB CPR and M-CPR could produce an adequate reduction in the anterior-posterior dimension of the chest. Therefore, the similar sternal displacements exhibited should produce cardiac compression to the same degree with either technique. The improved hemodynamic and respiratory parameters achieved by the LDB CPR over that by M-CPR might be explained by the collapse of medium-sized airways and air trapping in the lungs [4,20]. Airway collapse during CPR was evident under LDB CPR protocol, which increased the residual volume of the lung, together with more rapid decompression than that produced by M-CPR, increased venous return to the atrium. Another study demonstrated that the improvement in outcome following A-CPR due to its properties of increased contact area (A-CPR 232 cm^2^ versus M-CPR 39 cm^2^), which was capable of distributing the load over the chest wall and avoiding injuries [7].

There are two kinds of CPR in AutoPulse™ setting, namely A-CPR and C-CPR. According to AHA guideline, in the patient with patent airway, rescue breathing might perform continuously at 8-10/min simultaneous with continuous compressions. So we designed these three LDB CPR groups to investigate the effect of rescue breathing to hemodynamics during different LDB CPR protocols. Interestingly, the V-CPR group displayed lower AOD and CPP than the C-CPR group, which demonstrated that rescuing breathing simultaneous with continuous compressions might adverse to hemodynamics. This founding was consistent with the former study [21], which demonstrated that this adverse effect might due to the excessively high intrathoracic pressure induced by rescue breathing.

### Study limitations

The groups with rescue breathing received room air, while studies showed that rescue breathing by a bystander was 16% O_2_ (expired gas) [22,23] or if CPR was performed by a health provider, 100% O_2_ would be used. On the other hand, the shape of the porcine and human thorax is different and the heart and lungs are differently positioned relative to the sternum and thoracic wall, even the LDB that is scaled down from the original device to fit the porcine thoracic cage. Whereas all of these points are only of importance if extrapolating data and comparing the founding from piglets to humans, but should not bias the central aim of the study, which is comparing LDB to M-CPR.

## Conclusions

Compared with M-CPR, LDB CPR can significantly improve the hemodynamics and respiratory parameters during resuscitation, dramatically producing greater passive ventilation, which can improve gas exchange during CPR; together with the gasps, the volume of ventilation is adequate for maintaining gas exchange, so the rescue breathing during LDB CPR can be abridged.

## Abbreviations

CPR: Cardiopulmonary resuscitation; ROSC: Restoration of spontaneous circulation; LDB: Load-distributing band; TV: Tidal volume; RAP: Right atrial pressure; CO: Cardiac output; AOP: Aortic pressure; CPC: Cerebral performance category; CPP: Coronary perfusion pressure; AOD: Diastolic aortic pressure; RAD: Diastolic right atrial pressure; VD: Dead space; MValv: Alveolar minute volume.

## Competing interests

The authors declare that they have no competing interests. The manuscript, including related data, figures and tables, has not been published previously and that the manuscript is not under consideration elsewhere.

## Authors’ contributions

SW: conception and design of the research, drafting the manuscript and revising it critically for important intellectual content; JW: acquisition of data, analysis and interpretation of data; CL: giving final approval of the version to be published. “All authors read and approved the final manuscript.”

## Financial support

This study was supported by the Youth Science Foundation of Beijing Chaoyang Hospital, affiliated to Capital Medical University and the National Science Foundation of China (No.30972863).
